# Effect of *Medicago sativa* Addition on Physicochemical, Nutritional and Functional Characteristics of Corn Extrudates

**DOI:** 10.3390/foods10050928

**Published:** 2021-04-23

**Authors:** Marta Igual, Maria Simona Chiş, Sonia Ancuța Socaci, Dan Cristian Vodnar, Floricuța Ranga, Javier Martínez-Monzó, Purificación García-Segovia

**Affiliations:** 1 Food Investigation and Innovation Group, Food Technology Department, Universitat Politècnica de València, Camino de Vera s/n, 46022 Valencia, Spain; marigra@upvnet.upv.es (M.I.); xmartine@tal.upv.es (J.M.-M.); pugarse@tal.upv.es (P.G.-S.); 2 Department of Food Engineering, Faculty of Food Science and Technology, University of Agricultural Sciences and Veterinary Medicine of Cluj-Napoca, 3–5 Mănăştur Street, 400372 Cluj-Napoca, Romania; 3 Department of Food Science, Faculty of Food Science and Technology, University of Agricultural Sciences and Veterinary Medicine of Cluj-Napoca, 3–5 Mănăștur Street, 400372 Cluj-Napoca, Romania; sonia.socaci@usamvcluj.ro; 4 Faculty of Food Science and Technology, Institute of Life Sciences, University of Agricultural Sciences and Veterinary Medicine Cluj-Napoca, 3–5 Calea Mănăștur, 400372 Cluj-Napoca, Romania; dan.vodnar@usamvcluj.ro (D.C.V.); floricuta.ranga@usamvcluj.ro (F.R.)

**Keywords:** *Medicago sativa*, nutritional, functional, folates, phenolic acids, flavonoids

## Abstract

Currently, extrudates are considered unhealthy products, being characterized as high in calories; rich in fat, sugar, and salt; and low in nutritional compounds. The aim of this study was to assess the influence of lucerne (*Medicago sativa)* on the physicochemical, nutritional, and functional characteristics of corn extrudates. In order to achieve these goals, water content, water activity, expansion index, bulk density, porosity, hygroscopicity, optical properties, antioxidant activity, individual phenolic acids, folates, individual flavonoids, and volatile compounds were analyzed. The results showed that the typical characteristics of corn extrudates such as expansion, crunchiness, and density were maintained with 10% lucerne addition, highlighting a strong negative Pearson correlation (*p <* 0.05) between all studied parameters and lucerne addition. On the other hand, the lucerne addition caused a linear increase of bioactive compounds, showing positive Pearson correlations between flavonoids, folates, and antioxidant activity. The volatile profile of corn extrudates improved with the addition of lucerne leading to volatile compounds such as limonene, β-mircene, and hexanal. Overall, considering the textural, nutritional, and functional characteristics of corn extrudates, we determined that a percentage addition on 10% lucerne could be successfully used in the manufacturing of corn extrudates.

## 1. Introduction

Extrusion processing is an efficient technology applied to manufacture various food products such as snack foods, breakfast cereals, and pastas [1]. The consumption of ready-to-eat products obtained through extrusion have seen a substantial rise on the international market, mainly due to people’s lack of time [2]. Extrudates are defined as foods obtained through short-time thermal treatment at high temperatures and pressures, under continuous stirring [3]. Corn flour is one of the most common cereals used to manufacture expanded extrudates [4]. From a nutritional viewpoint, corn does not fulfill all the consumers requirements [4], and has been called a “poor man’s nutricereal” [5]. To overcome this nutritional drawback, several raw vegetable materials are used.

Lucerne (*Medicago sativa*; MS), also known as alfalfa, is a valuable perennial legume that has gained the researchers attention due to its valuable nutritional composition, being a rich source in protein, amino acids, phytochemical substances (L-canavanine, isoflavones, coumestrol, chlorophyll, and carotene), vitamins, folate, and minerals such as iron and calcium [6,7,8]. Phytoestrogens (isoflavones, lignans, and coumestans) are biomolecules that have been identified in MS as having a positive influence on human health, being involved in the prevention of different diseases such as osteoporosis, cardiovascular diseases, menopausal symptoms, but also in cancer chemoprevention and therapy [9]. Due to its content in flavonoids, MS showed positive effects in the apoptosis and cytotoxicity of human erythroleukemia and mammary adenocarcinoma [10]. The MS pharmaceutical effects are also highlighted in the prevention of anemia, diabetes, low bone density, endometriosis, and stomach ulcers [11]. MS has been called “the queen of forages” thanks to it being rich in bioactive compounds, low in cholesterol content, and an inexpensive source of protein.

Notably, extracts from MS could have antibacterial, antifungal, and nematocidal effects and MS has been used for centuries in folk medicine to treat asthma, arthritis, and nervous system disorders [8,12]. Currently, it is mainly used for animal food, but it could also be used as a raw material in human nutrition such as in salads, soups, puddings, tortillas, sandwiches, croquettes, or as food supplements [11,13]. Specifically, the European Food Safety Authority has integrated MS as a dietary supplement [13], and since 2009, lucerne concentrates have been allowed in human nutrition [6].

In the UK, crisps and crisp-style snacks are staple foods, consumed in 90% of the households. Many campaigns encourage the reduction of snack calories and sugar content, especially in children’s diets. As reported by Grasso [14], children’s sugar consumption is three times higher than the daily recommended intake; further, there is a need to increase adult daily fiber content. Moreover, the nutritional value of snacks is low, they are rich in fat and salt [14] and low in protein and amino acids [15,16]. Today, high-value food products have attracted broad attention and the ready-to-eat food industry is focused on developing new healthy foods. The consumption of snacks is important for our diet because it allows us to achieve a good fractionation, control the level of hunger or appetite, and keep the digestive system active; however, its formulations should be directed toward healthier products. The snack fortification trend with enriched vegetable sources has gained researchers’ interest, with the aim of improving the nutritional value of ready-to eat products. Thus, the aim of this study was to evaluate the effects of enrichment with varied quantities of MS powder on the nutritive and functional values, physicochemical properties, and extrusion parameters of extruded snacks.

## 2. Materials and Methods

### 2.1. Standards and Reagents

The pure standard of folic acid was purchased from Supelco (Sigma-Aldrich, St. Louis, MO, USA) and the pure standards of chlorogenic acid (>98% HPLC), luteolin (>99% HPLC), and gallic acid (>99% HPLC) were purchased from the same supplier. Acetonitrile and acetic acid HPLC-gradient were provided by Merck (Darmstadt, Germany), and water was purified with a Millipore Direct-Q UV system from Merck (Darmstadt, Germany).

### 2.2. Raw Materials

Corn grits (CM) were purchased from Maicerías Españolas S.L. (Valencia, Spain); MS powder was purchased from Inkanatuta Import Export S.L. (Donostia-San Sebastián, Spain).

### 2.3. Formulations and Extrusion Processing

**Figure 1:**
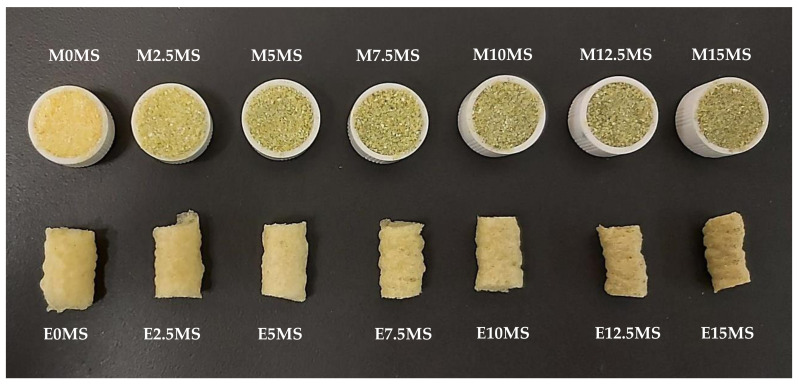
Appearance of studied mixtures (M) and extrudates (E), with different concentrations (0, 2.5, 5, 7.5, 10, 12.5, and 15%) of *Medicago Sativa* (MS).

CM (200 g) was mixed manually using a whisk, with increasing MS powder percentages of 2.5, 5, 7.5, 10, 12.5, and 15% to produce the extrusion mixtures. The particle sizes of CM and MS were 511 µm and 163 µm, respectively. The CM and MS visual colors are shown in Figure 1. CM could be characterized as having a pale yellow color and MS as having a pleasant green color. Extrusion was performed using a single-screw laboratory extruder (Kompaktextruder KE 19/25; Brabender, Duisburg, Germany) with a barrel diameter of 19 mm and a length/diameter ratio of 25:1. The extruder was operated at a 3:1 compression ratio, loaded with prepared corn samples at a constant dosing speed of 18 rpm (feed rate range, 3.4 kg/h). The screw was rotated constantly at 150 rpm, and the temperatures of barrel sections 1–4 were set to 25, 70, 170, and 175 °C, respectively; the nozzle diameter was 3 mm. Motor torque, screw speed, barrel temperatures, and melt pressure were monitored using Extruder Winext software (Brabender). Calculated specific mechanical energy of the corn extrusion ranged from 900 to 1100 kJ/kg. The pressure measured on the extruder head ranged between 112 and 222 bar. Extrudates were cooled at ambient temperature and sealed in plastic bags for further analysis.

### 2.4. Analysis

#### 2.4.1. Water Content and Water Activity

Water content (x_w_) (g water/100 g sample) was determined using vacuum oven drying at 105 °C until a constant weight was obtained [17] for mixtures with and without MS percentage addition and extruded samples. Samples were analyzed in triplicate. Water loss, because of the extrusion process, was calculated. Water activity (a_w_) of the extruded samples was determined by the AquaLab PRE LabFerrer equipment (Pullman, Washington, DC, USA).

#### 2.4.2. Expansion Index (SEI)

The diameter of 20 extruded product pieces was measured for each sample with an electronic Vernier caliper (Comecta S.A., Barcelona, Spain). The surface expansion index of the die (SEI) was determined as the quotient between the square of the measured diameters and the square of the die diameter [18].

#### 2.4.3. Bulk Density and Porosity

The porosity (ε), the percentage of air volume related to the total volume, was calculated from the true (ρ) and bulk (ρ_b_) densities according to García-Segovia et al. [19] and Equation (1).

(1) $$\varepsilon =\frac{\left(\rho -\rho_{b}\right)}{\rho }$$

The real density of the extruded products was determined using a helium pycnometer (AccPyc 1330, Micromeritics, Norcross, GA, USA). For bulk density (ρ_b_) determination, measurements were taken 15 times, where the height and diameter of cylinders were measured with an electronic Vernier caliper (Comecta S.A., Barcelona, Spain) and samples were weighed with a precision scale (±0.001 g) (Mettler Toledo, Greifensee, Switzerland).

#### 2.4.4. Water Absorption Index, Water Solubility Index, and Swelling Index

The water solubility index (WSI) and water absorption index (WAI) were determined by the method of Singh and Smith [20]. The extrudates were first milled to a mean particle size of 180–250 µm. A 2.5 g sample was dispersed in 25 g of distilled water, using a rod to manually break up any lumps. After stirring for 30 min using a magnetic stirrer, the dispersions were rinsed into tared 50 mL centrifuge tubes, made up to 32.5 g and centrifuged at 3000× *g* for 10 min. The supernatant was decanted for determination of its dissolved solids content, and the sediment was weighed. WAI and WSI were calculated according to Uribe-Wandurraga et al. [21] and Equations (2) and (3).

(2) $$\mathrm{WAI}\;=\frac{\mathrm{weight}\;\mathrm{of}\;\mathrm{sediment}\;}{\mathrm{weight}\;\mathrm{of}\;\mathrm{dry}\;\mathrm{solids}\;}$$

(3) $$\mathrm{WSI}\;\left(\%\right)=\left(\frac{\mathrm{weight}\;\mathrm{of}\;\mathrm{dissolved}\;\mathrm{solids}\;\mathrm{in}\;\mathrm{supernatant}\;}{\mathrm{weight}\;\mathrm{of}\;\mathrm{dry}\;\mathrm{solids}}\right)\times 100$$

The swelling index (SWE) was measured using the bed volume technique. Samples were accurately weighed (1 g) and transferred to a calibrated cylinder and 10 mL of distilled water was added. Cylinders were left to stand undisturbed for 18 h at ambient temperature. The bed volume was recorded and expressed as mm of swollen sample per g of dry initial sample [22].

#### 2.4.5. Hygroscopicity

Approximately 0.5 g of each extruded sample were placed in a petri dish at 25 °C in an airtight plastic container containing a Na_2_SO_4_ saturated solution (81% relative humidity). After 1 and 7 days each sample was weighed and the hygroscopicity (Hy) was expressed as g of water gained per 100 g dry solids [23].

#### 2.4.6. Texture

The texture of extrudates, using puncture tests, was measured using a TA-XT2 Texture Analyzer (Stable Micro Systems Ltd., Godalming, UK) and Texture Exponent software (Version 6.1.12.0,Godalming, Surrey, UK) [24]. A 2 mm diameter cylinder was used, and the crosshead speed was kept at 0.6 mm/s; measurements were taken 10 times. The software recorded and analyzed the force–time curve, and gave an area under the curve plot, which represented work done (S) for a time of displacement (t) of the puncturing device. The force-drop (F) of each peak represented the local resistance of cell walls, the number of peaks (N_o_) were also recorded. These parameters were used to calculate the average puncturing force (F_p_), average specific force of structural ruptures (F_s_), spatial frequency of structural ruptures (N_sr_), and crispness work (W_c_) according to Bouvier [25] and Igual et al. [3].

#### 2.4.7. Optical Properties

Translucency and CIE*L*a*b* color coordinates were determined from the surface reflectance spectra obtained between 400 and 700 nm, when measuring on white and black backgrounds, considering a standard light source D65 and a standard observer 10° (Minolta spectrophotometer CM-3600d, Japan). Measurements of the extruded samples were taken 10 times. The translucency of the samples was determined by applying the Kubelka–Munk theory for multiple scattering of the reflection spectra [26]. This theory assumes that the light flux passing through the sample is related to the ratio of absorbed versus scattered light. The calculated reflectance of an infinitely thick layer of the material was used to obtain the coordinates CIE*L*a*b, hue (h*), and chroma (C*) color attributes. The total color differences of mixtures or extrudates with MS (ΔE_1_) were calculated for the control sample. To evaluate the color changes of the mixtures because of extrusion, total color difference (ΔE2) was calculated between each mixture extruded at the same MS percentage addition.

#### 2.4.8. Antioxidant Capacity

Antioxidant capacity (AC) was assessed using the free radical scavenging activity of the samples evaluated in triplicate with the stable radical 2,2-diphenyl-1-picryl-hydrazyl-hydrate (DPPH) following Agudelo et al.’s [27] methodology. A UV-visible spectrophotometer (Helios Zeta, Thermo Electron Corporation, Loughborough, LE, UK) was used to test the absorbance at 515 nm. The results were expressed as milligram Trolox equivalents (TE) per 100 g (mg TE/100 g).

#### 2.4.9. Extraction and Analysis of Phenolic Compounds by HPLC/DAD/ESI-MS

The extracted samples were made by using ultrasound-assisted extraction (UAE). Briefly, 0.5 g of sample was mixed with 2 mL methanol and 1% HCl, vortexed for 1 min with a vortex Reax top (Heidolph, Schwabach, Germany) and ultrasonicated for 30 min using an ultrasonic bath (Elmasonic E15H, Elma, Singen, Germany). After centrifugation (4000× *g* for 10 min) with an Eppendorf 5804 centrifuge (Eppendorf, Hamburg, Germany), the supernatants were filtered through a 0.45 µm nylon filter (Millipore, Merck KGaA, Darmstadt, Germany), and 20 µl were used for further analysis.

The identification and characterization of the individual phenolic compounds was made according to Călinoi and Vodnar [28] and conducted using an HP-1200 liquid chromatograph equipped with a quaternary pump, autosampler, DAD detector, and MS-6110 single quadrupole API-electrospray detector (Agilent Technologies, Santa Clara, CA, USA). An XDB C18 Eclipse column (4.5 × 150 mm, 5 mm) (Agilent Technologies) was used for the separation of the phenolic compounds, at a temperature of 25 ± 0.5 °C. The mobile phase (A) comprised water acidified by acetic acid 0.1% (*v*/*v*) and phase B was made with acetonitrile acidified by acetic acid 0.1% (99:1, *v*/*v*) with the flow rate of 0.5 mL/min. The following multistep linear gradient was applied: start with 5% B for 2 min; increase from 5% to 90% B in 20 min, hold for 4 min at 90% B, then take 6 min to arrive at 5% B. Total time of analysis was 30 min, flow rate 0.5 mL/min, and oven temperature 25 ± 0.5 °C. Mass spectrometric (MS) detection of positively charged ions was performed using the Scan mode. For the MS fragmentation a scanning range of 100–1200 *m*/*z* in the ESI (+) mode was applied and the capillary voltage and the nitrogen flow were set at 3000 V, 350 °C, and 7 L/min, respectively.

Spectra values were detected in a range between 200 and 600 nm and DAD was used to monitor the eluent. The detections were registered at λ 254, 280, and 340 nm. Agilent ChemStation software (Rev B.04.02 SP1, Palo Alto, California, USA) was used to acquire and analyze the samples. Calibration curves with luteolin (r^2^ = 0.9972), gallic acid (r^2^ = 0.9978), and chlorogenic acid (r^2^ = 0.9937) were used to quantify the amount of flavonoids (flavones and isoflavones), hydroxybenzoic acids, and hydroxycinnamic acids, respectively.

#### 2.4.10. Extraction and Analysis of Folate through HPLC/DAD/ESI-MS Assay

The sample extraction was fulfilled by mixing 1 g of sample with 5 mL of phosphate buffer (pH = 7), sonicating for 30 min using an ultrasonic bath (Elmasonic E15H, Elma, Singen, Germany), centrifuging at 4000× *g* for 10 min at 24 °C, filtering the supernatant, and injecting it in the HPLC system.

The analysis of total folate was made by using a HP-1200 liquid chromatograph equipped with a quaternary pump, autosampler, DAD detector, and MS-6110 single quadrupole API-electrospray detector (Agilent Technologies). The positive ionization mode was applied to detect the folic acid; different fragmentors, in the range 50–100 V, were applied. The column was an Eclipse XDB-C18 (5 μm; 4.5 × 150 mm i.d.; Agilent Technologies). The mobile phase was acetonitrile/acetic acid 1% in a ratio of 20/80 (*v*/*v*) in the isocratic system. The total time of analysis was 10 min, flow rate was 0.5 mL/min, and oven temperature was 25 ± 0.5 °C. Mass spectrometric detection of positively charged ions was performed using the Scan mode. The applied experimental conditions were gas temperature 350 °C, nitrogen flow 7 lL/min, nebulizer pressure 35 psi, capillary voltage 3000 V, fragmentor 100 eV, and *m*/*z* 120–600. Chromatograms were recorded at wavelength λ = 280 nm and data acquisitions were conducted with the Agilent ChemStation software (Rev B.04.02 SP1).

#### 2.4.11. Analysis of Volatile Compounds by ITEX/GS-MS

The extraction and analysis of volatile compounds was performed according to Chis et al. [29] using the in-tube extraction technique (ITEX) followed by separation and identification of the compounds by gas chromatography-mass spectrometry (GC-MS), using a GC-MS QP-2010 model (Shimadzu Scientific Instruments, Kyoto, Japan) equipped with a Combi-PAL AOC-5000 autosampler (CTC Analytics, Zwingen, Switzerland) and a capillary column (ZB-5 ms, 30 m × 0.25 mm i.d. × 0.25 µm, Phenomenex, Torrance, CA, USA). For the volatile extraction step, a hermetically sealed headspace vial containing 0.5 g of sample was incubated at 60 °C under continuous agitation for 10 min. After the incubation, from the headspace phase, the volatile compounds were adsorbed (aided by the headspace syringe) repeatedly (15 strokes) into a porous polymer fiber microtrap (ITEX-2TRAPTXTA, Tenax TA 80/100 mesh, ea). The extraction of the volatile compounds and their thermal desorption and injection into the GC-MS injector were performed automatically using the Combi-PAL AOC-5000 autosampler. The parameters for the column oven were from 38 °C the temperature was increased to 110 °C and then to 250 °C at 4 °C/min and 20 °C/min, respectively, and the final temperature was held for 5 min. The identification of the samples’ volatiles was based on their mass spectra using the software’s NIST27 and NIST147 mass spectra libraries, and they were verified and compared with retention indices drawn from flavornet and pherobase databases [30,31]. The odor perception for each identified compound is specified in Table 6 according to the information found in the aforementioned databases [30,31]. The results are expressed as relative percentage from total peaks area.

### 2.5. Statistical Analysis

Analysis of variance (ANOVA), with a confidence level of 95% (*p* < 0.05), using Statgraphics Centurion XVII Software, version 17.2.04, was applied to evaluate the differences among mixtures or extruded samples and to evaluate the extrusion process. The method used to discriminate between means was Fisher’s least significant difference procedure. A correlation analysis among the extrusion parameters and textural properties of produced extrudates, with a 95% significance level, was conducted (Statgraphics Centurion XVII). Pearson correlation also was applied to explore the relationship between antioxidant capacity and the analyzed compounds.

## 3. Results and Discussion

### 3.1. Physicochemical Characteristics of Extrudates

Table 1 shows mean values and standard deviations of x_w_, a_w_, WAI, WSI, SWE, Hy, SEI, ρ_b_, and ε of extrudates. Even if MS addition decreases extrudates x_w_, no significant (*p* > 0.05) differences were observed among samples, meanwhile, a significant difference (*p* < 0.05) was noticed between control extrudate (0% MS addition) and samples. Water content and water activity of extruded snacks were in the range of low moisture foods [32]. In addition, the values of x_w_ and a_w_ were like other corn snacks obtained by Igual et al. [3]. Extrusion moisture loss was 9% in the control (without MS) and ranged from 20 (2.5, 5, 7.5, and 10% MS) to 30% (12.5 and 15% MS) in samples with MS. Moisture loss at the die depends on the vapor pressure inside the air cells and matrix characteristics such as extensibility and water binding. It reflects the degree of starch transformation; ungelatinized starch has poor film forming ability, its presence reduces extensibility, and residual water is trapped inside the structure rather than escaping at vapor flashpoint. Other poorly extensible biopolymers may have the same effect on moisture loss (such as insoluble fiber) [33]. Furthermore, it should be mentioned that MS is a rich source in fiber, the content of which varies between 3.5 and 27.3 g/100 g, for green forage to leaf meal, respectively [34], and, therefore, could be involved in the moisture loss.

WAI and WSI showed an opposite trend, WAI increased significantly (*p <* 0.05) with the MS addition whereas WSI decreased significantly (*p <* 0.05) with the MS addition. This opposite trend could be justified by the MS and CM chemical composition, mainly fiber and starch. The increased MS percentage, which contains a high fiber amount [34] decreased the CM starch content, enhancing the WAI values and decreasing the WSI value. These two indices show how extrudates interact with water [35]. WAI indicates the portion of water absorbed by the extrudate when immersed in water [36], whereas WSI indicates the water solubilized components released during extrusion that can cause molecular damage [37]. Therefore, MS in extrudate mixtures reduces the risk of possible molecular damage by molecules solubilized in water. This indicates that lucerne causes less solubilization of matrix components during extrusion. Control samples presented the lowest values of SWE, thus the lowest capacity to swell and thus to satiate [38]. SWE values increased significantly (*p <* 0.05) with MS addition, this increase was higher with 10–15% MS addition. Hy increased significantly (*p <* 0.05) when MS % was increased in mixtures; however, samples with 7.5–15% MS did not show significant (*p >* 0.05) differences among them.

Expansion takes place because of the sudden exit of molten mass from the restricted die, from an area if high pressure to atmospheric pressure, giving improved texture to the extrudate. It depends on the feed composition, extent of cooking, and melt flow in the die. Expansion at the die defines the structure, and thus it is important to optimize the process [25]. In this study, SEI values decreased from 14.6 (without MS) to 6.9 (with 15% MS). MS addition in mixtures provoked a significant (*p <* 0.05) decrease in SEI at any concentration. Losing expansion with an increase of MS percentages could be because the fiber prevents the air molecules from expanding [39].

Density is a general property of the extrudate, which indicates overall expansion and changes in material parameters and cell structure, pores, and voids developed as the result of processing; highly expanded extruded materials show a porous structure [40]. Bulk density of extrudates describes the degree of puffing experienced by the material as it exits the extruder, it considers expansion in all directions, different to SEI that considers expansion only in the direction perpendicular to the extrudate flow [41]. There were significant (*p <* 0.05) differences in ρ_b_ values: Extrudates with 15% MS showed the highest values, because the same volume weighs more than the others. However, incorporating MS in mixtures decreased ε. When extrudates contained 5–15% MS, ε was maintained around 86%, independent the MS % percentages inside the range. ε values of the control sample (without MS) was similar to other corn extrudates [21].

The texture of the extrudate is an important physical property for ready-to-eat snack products, which largely depends on the composition of the raw material of the mix used for extrusion [40]. Table 1 shows mean values and standard deviations of textural parameters of extrudates. Crispness work (W_c_) can be interpreted as the sensory parameter of fracturability and describes the work required to fracture one pore or a group of pores. Average puncturing force (F_p_) and average specific force of structural ruptures (F_s_) of extruded products is usually associated with the sensory perception of hardness during chewing—defined as the force to compress a solid substance between the molar teeth [42]. Spatial frequency of ruptures (N_sr_) describes the number of fracture events during puncture and (N_0_) is the number of fractures along the assay. W_c_ was significantly (*p <* 0.05) greater in samples with 12.5 and 15% MS than in extrudates with 0–10% MS. Extrudates with a high concentration of MS (12.5 and 15%) were significantly (*p <* 0.05) less crunchy (N_0_) than the others and presented the highest hardness (F_s_ and F_p_) because of a higher density of these samples. Extrudates within MS percentages ranging from 2.5% to 10% showed higher N_sr_ values than did the rest of the samples. Other authors indicate there is a positive correlation between N_sr_ and pore size of extruded snacks [43]. Chanvrier et al. [44] related this behavior to the propagation of fractures at the phase interface: large pores exhibit a greater interfacial area, giving more data peaks.

Pearson correlation coefficients among studied parameters in Table 1 and MS percentages were conducted (Table 2). There were significant (*p <* 0.05) correlations among MS percentages and all studied parameters except for N_sr_. When MS % increased, WAI, SWE, Hy, and ρ_b_ increased whereas x_w_, a_w_, WSI, SEI, and ε decreased. WSI presented the highest Pearson coefficient with MS percentages. The a_w_ showed a high correlation with MS %, along with WAI and SEI. Water content and water activity presented significant (*p <* 0.05) positive correlations with SEI and ε whereas a_w_ and x_w_ correlated negatively with ρ_b_. Thus, greater expanded snacks will be wetter, because the higher the water content, the lower is the resistance to bubble growth [45]. The a_w_ and x_w_ were also correlated negatively with WAI, SWE, and Hy, and positively with WSI. Significant (*p <* 0.05) negative correlations among Hy with SEI and ε, and positive correlations obtained with ρ_b_ demonstrated that a highly hygroscopic product has low water content, is poorly expanded and porous, but has a high bulk density. Therefore, the greater the incorporation of MS, the longer the shelf life of the product and the lower the risk of molecular damage, but its expansion will be less. Expansion properties correlated with each other: high SEI leads to high ε and low ρ_b_, and vice versa, which corroborates other studies [19,39].

Related to textural parameters, higher MS % in mixtures results in greater hardness (F_s_ and F_p_) and crispness work (W_c_) in extrudates and lower (N_0_). All texture parameters were significantly (*p* < 0.05) correlated with ρ_b_ and F_p_, and F_s_ presented the highest Pearson correlation. Hence, samples with high ρ_b_ exhibited greater hardness, because of a larger compaction of the extruded products. Many studies have reached the same results by relating hardness to bulk density [46]. The increased values of hardness in extrudates could be also justified by the MS chemical composition, mainly in protein and fiber contents. For instance, Giuberti et al. [47] highlighted that hardness could be influenced by water–starch–protein interactions but also by fiber content of the raw materials used in the products manufacturing. In the present study, the MS protein content could be involved in the formation of a harder extrudates structure, being a result of the strong adherence of protein and starch. Furthermore, fiber could influence the extrudates hardness, contributing to the extrudates structure compaction.

W_c_, F_s_, and F_p_ showed a significant (*p <* 0.05) positive correlation with WAI and a negative correlation with x_w_, a_w_, and WSI. Thus, the greater the WAI of a product, the more resistant it is to fracture, therefore, the harder it will be [19]. Crunchiness (N_0_) presented significant (*p* < 0.05) positive correlations with WSI; meanwhile, it was not positively correlated with a_w_. According to Lotfi Shirazi et al. [48], the crunchiness of snacks is related to the thickness of the walls and with expansion, available water inside the product is necessary for its starch binding and thus to facilitate its gelatinization and expansion [49]. The significant (*p <* 0.05) positive correlation between SEI and x_w_ and a_w_ can be observed.

Optical properties of mixtures and extrudates are included in Table 3 (L*, a*, b*, C*, h*, and ΔE). Both mixtures and extrudates with or without MS did not show differences in the measurements taken on white and black backgrounds; therefore, they were not translucent and color coordinates CIE*L*a*b* and the values of chroma (C*) and tone (h*) were obtained directly from the equipment used for color measurement. In contrast, other studies [3,19] showed differences in those measurements (white and black backgrounds) in corn extrudates. MS addition decreased mixtures L*, a*, b* and C* significantly (*p <* 0.05) and increased the mixtures’ h*. Moreover, there were significant (*p >* 0.05) differences among samples as a function of MS percentage. Samples with MS were less whitish and more blue-greenish. Total color differences (ΔE_1_) between samples with MS and the control ranged between 13 and 18, higher than 3 units; therefore, humanly perceptible [50]. The extrusion process provoked significant (*p <* 0.05) changes in color coordinates and total color differences. L*, b*, C*, h*, and ΔE decreased whereas a* increased. Color is an important quality parameter because it reflects the extent of chemical reactions and degree of cooking or degradation that take place during the extrusion process. ΔE_2_ represents the total color difference compared to the color of mixtures. Higher ΔE_2_ means darker products with more intense yellow and red color. This was also observed by Dogan et al. [51] in quinoa extrudates.

The total color change in extruded products ranged between 6.3 and 32.2. These ΔE_2_ values were significantly higher in control extrudates (without MS) than in the rest of the extrudates. Higher MS percentages in extrudates resulted in significantly (*p <* 0.05) lower L* and higher b*, C*, and ΔE_1_. Figure 1 shows the appearance of the mixtures and extrudates. In concordance with color coordinates, the greenish color (a* decrease) of the mixture with increasing MS % is remarkable. Moreover, extrudates lost the greenish color compared to mixtures, as is observed in ΔE_2_. However, the reduction of the extrusion expansion with MS % increase can be perceived.

### 3.2. Nutritional and Functional Value of Extrudates

#### 3.2.1. Antioxidant, Phenolic Acids, Folates, and Flavonoids Content

MS is a rich source of flavonoids, polyphenolic compounds with highly antioxidant properties that act as natural fungicides and insecticides. The main flavonoids classes are divided into flavonols, flavones, isoflavones, flavanones, aurones, chalcones, and anthocyanidins [12]. In this study, the MS AC showed a total amount of 855 (41) µg TEq/g d.w. (data not shown) and could be explained by the higher amount of flavonoids, which might possess antioxidant properties [12].

Table 4 shows the mean values and standard deviations of phenolic acids, folates, and antioxidant activity of mixtures and extrudate samples with different MS percentages. Mixture antioxidant activity increased linearly with increasing levels of MS, leading to a final value of 358 (9) µg TEq/g d.w., highlighting its strong antioxidant activity. Furthermore, the extrusion process caused a significant decrease (*p <* 0.05) of extrudates’ antioxidant activity amount compared to that of the mixtures, as showed in Table 4. This could be explained as critical factors could decrease the polyphenolic compounds and therefore the AC. Screw speed and temperature are critical factors that could have a negative influence on the amount of bioactive compounds, as reported by Brennan et al. [52]. Likewise, Wani and Kumar [53] showed that during an extrusion cooking process, snack antioxidant activity and phenolics contents decreased, and Korus et al. [54] highlighted that during extrusion the antioxidant activity of the extrudates decreased compared to that of the raw materials. Even if the extrusion process significantly decreased (*p <* 0.05) the amount of the mixtures’ antioxidant activity compared with extrudates, in extrudates samples, adding MS had a significant positive influence on the antioxidant activity (*p <* 0.05).

Sample mixtures highlighted an important number of phenolic acids (Table 4), of which p-coum and Di-caff showed higher extended values. Due to the MS addition in mixtures, a slight decrease of phenols amount was observed, but at 15% MS, the total content of identified phenols was still higher, reaching a total value of 1178.62 μg/g d.w. and being statistically different from that of the other mixtures (*p <* 0.05).

However, the extraction of phenolic compounds could be improved with the extrusion process through disintegration of the cell wall matrices and breaking of the complexed polyphenols with high molecular weight [55]. Therefore, even if MS is not a reliable source of phenolic acids, the phenolic acids content was more stable during the extrusion process, or through extrusion their amount increased (*p <* 0.05) (Table 4). For instance, the Di-caff acid and Fer phenolic acid mixtures increased their amount during extrusion, reaching values of 57.22 µg/g d.w. and 40.22 µg/g d.w., starting from initial values of 47.06 µg/g d.w. and 31.37 µg/g d.w in mixtures, respectively.

This is in line with Arribas et al. [56] who showed that organic acids from rice and legumes were not significantly affected by the extrusion process and with Cruz et al. [57] who reported an increase of phenolic acids during extrusion.

The MS exhibited higher folate content than did corn grits, reaching a value of 24.40 μg/g d.w. compared to 0.83 μg/g d.w., respectively. Folic acid and its derivates are included in the generic description of folate, which plays an essential role in neural tube development and in lung carcinogenesis [58].

Conversely, the amount of folates showed a seemingly proportional effect with the addition of MS, reaching a final value of 4.86 μg/g d.w for the 15% MS mixture, starting from a value of 0.83 μg/g d.w for the 0% MS mixture (Table 4). This could be explained by the MS-rich folates content. Regarding the extrudates folate content, adding MS positively influenced their content (Table 4), 15% MS extrudates had a final value of 4.18 μg/g d.w. During the extrusion process of 15% MS sample, the folates ccontent decreased by 16% percentages.

The Council directive on the 24 September 1990 on nutrition labeling for foodstuffs [59] in the annex, specify that the recommended daily allowance of folate is 200 µg. According to this directive and the regulation no. 1924/2006 of the European Parliament and of the Council of 20 December 2006 on nutrition and health claims made in foods [60], extrudates enrichment with MS are a food “high in folate” at all studied MS percentages. Moreover, the intake of 74 g of corn extrudate with 12.5 MS percentages can give the recommended daily allowances of folate. With the extrudates with 15% MS and with an intake of 50 g, the recommended daily allowances of folate quantities are reached.

Regarding MS flavonoids content, four isoflavones and eight flavones were identified (Table 5). The main isoflavone was represented by Genistein-glucoside (G-gluc), having a total amount of 1000.63 μg/g d.w., whereas the main flavone was Apigenin-(feruloyl-glucuronyl)-glucuronide (A-fer-gg), with a total amount of 3164.14 μg/g d.w.

Adding different percentages of MS in mixtures had a positive influence on the flavones and isoflavones content (Table 5) because of the enriched content of flavonoids in MS. Moreover, A-fer-gg and A-fer-ggg increased linearly with increasing levels of MS (*p <* 0.05), reaching final values of 552.06 μg/g d.w and 77.90 μg/g d.w., respectively. Regarding isoflavones content, adding 15% MS caused an increase of all isoflavones, the biggest amounts being recorded by Genistein-glucoside (Genistin), G-gluc, and Daidzein (D).

Regarding the flavonoids content of the extrudates (Table 5), Apigenin-glucoside (A-gluc), genisteis (G), Apigenin-glucuronide (A-glucur), and Apigenin-diglucuronide (A-diglucur) were lost during the extrusion process. However, addition of 15% MS caused a significant increase (*p <* 0.05) of A-fer ggg, A-fer gg, L-diglucur, and Liquiritigenin (Liq-gen), whereas from the isoflavones group, only Genistein (G) was lost during the extrusion process.

The decrease or loss of flavones and isoflavones during the extrusion process could be explained by the decomposition and polymerization of heat-labile phenolic and phenolic compounds, respectively. Furthermore, a higher temperature could positively influence the phenolic acid oxidation, leading to their reduced amount [55]. A negative high temperature influence on the phenolic compounds content during extrusion was also reported by Sarawong et al. [61]. Moreover, Ramos Diaz et al. [62] explained that the extractability of phenolic compounds could be affected by the protein complexation because of its denaturation during the extrusion process.

To explain the relationship of the different compounds quantified in this study with the AC of the samples, and among them, correlation statistical analyses were performed. Studied flavonoids and folates showed a positive Pearson’s correlation coefficient with AC. Folates, A-fer-ggg, and D played a significant role in the AC of mixtures and extrudes showing 0.8935, 0.8690, and 0.8573 (*p <* 0.05) values of the Pearson coefficient, respectively.

#### 3.2.2. Volatile Compounds from Extrudates

Table 6 shows that MS is a rich source of volatile compounds. We identified 37 compounds: 4 alcohols, 5 aldehydes, 19 terpenes and terpenoids, 1 acid, 3 ketones, 1 hydrocarbon, and 4 esters. The main volatile compounds from MS were the group of terpenes and terpenoids, from which limonene (45.34%), beta-myrcene (21.78%), and *p*-cymene (8.55%) reached the highest extended values. These compounds gave to MS an odors perception like citrus, mint, balsamic, must, spice and citrus, herbal, and sweet, respectively (Table 6). Table 7 shows the mean values and standard deviations of volatile compounds from mixtures and extrudate samples. The main volatile compounds from mixtures with 15% MS were represented by the terpenes and terpenoids group, limonene (38.85%), β-myrcene (25.10%), and *p*-Cymene (10.21%). This could be related to the large amount of volatile compounds in the raw materials.

The raw materials used during extrusion processes can undergo several chemical transformations such as protein denaturation, complex formation between amylose and lipids, and starch gelatinization [63]. Regarding the formation of volatile compounds during an extrusion process, reactions such as Maillard and lipid degradation are involved. Aroma compounds such as aldehydes could be formed through the interactions between reducing sugars and amino acids [64]. Strecker aldehydes are one of the main volatile compounds because of decarboxylation and deamination of amino acids through the Maillard reaction [65]. The amino acids are important precursors, enhancing the flavor compounds through the Maillard reaction [64] at temperatures higher than 130 °C [66]. Therefore, MS is a valuable source of amino acids, having a total amount higher than those identified in eggs or wheat [6], which could be involved in flavor compound development.

Hexanal was the main volatile compound from the aldehydes group, having the highest peak area in all samples, followed by limonene, β-myrcene, and eucalyptol. The amount of hexanal was statistically different when comparing its amount in mixtures to that in extrudates (*p <* 0.05).

However, Lampi et al. [66] stated that hexanal might be the major product resulting from the autoxidation of linoleic acid through decomposition of 13-hydroperoxides and could be better released through the extrusion process.

Limonene content was lower in extrudates than in the mixtures, and this could be because of the influence of extruder torque and the extrudate expansion ratio. Even if the amount decreased, at 15% MS the limonene peak was still higher (29.17%). This could be due to the interaction between limonene and starch, through the inclusion of complexation, according to Yuliani et al. [67].

## 4. Conclusions

The increased MS percentages influenced in a positive way the low expansion and crunchy extrudates, lowering the risk of possible molecular damage by molecules solubilized in water and, therefore, making them more stable. Furthermore, in order to maintain the typical extrudate characteristics, a maximum MS percentage of 10% is highly recommended. From the nutritional point of view, the corn extrudates’ antioxidant activity, flavones (L-fer-ggg, A-fer-gg, A-fer-ggg, and L-diglucur), isoflavones (Liq-gen, D-gluc, and G-gluc, D), and folates amounts increased linearly with increasing levels of lucerne, leading to products enriched in bioactive compounds. Moreover, the intake of only 74 g of corn extrudate with 12.5 MS addition could ensure the daily folate recommendation. The aroma compounds of mixtures and extrudates showed a positive feedback loop of their odor perception. Therefore, from the physicochemical, nutritional, and functional point of views, using MS to manufacture corn extrudates is highly recommended at a maximum concentration of 10%.

## Figures and Tables

**Table 1:** Mean values (and standard deviations) of water content (x_w_), water activity (a_w_), water absorption index (WAI), water solubility index (WSI), swelling index (SWE), hygroscopicity (Hy), expansion index (SEI), bulk density (ρ_b_), porosity (ε), crispness work (W_c_), spatial frequency of structural ruptures (N_sr_), average specific force of structural ruptures (F_s_), average puncturing force (F_p_), and number of peaks (N_0_) of extrudates with increasing MS powder percentages.

MS (%)	0	2.5	5	7.5	10	12.5	15
x_w_ (g_w_/100 g)	5.76 (1.12) ^a^	4.3 (0.2) ^b^	4.11 (0.18) ^b^	3.99 (0.08) ^b^	3.97 (0.08) ^b^	3.29 (0.12) ^b^	3.36 (0.21) ^b^
a_w_	0.366 (0.003) ^a^	0.365 (0.003) ^a^	0.364 (0.003) ^a^	0.347 (0.003) ^b^	0.338 (0.003) ^c^	0.331 (0.003) ^d^	0.330 (0.003) ^d^
WAI	3.96 (0.02) ^f^	4.40 (0.03) ^e^	4.62(0.03) ^d^	5.39 (0.03) ^b^	5.17 (0.13) ^c^	5.31 (0.06) ^b^	5.86 (0.03) ^a^
WSI (%)	19.0 (0.2) ^a^	16.83 (0.05) ^b^	13.42 (0.13) ^c^	8.16 (0.04) ^d^	8.1 (0.2) ^d^	6.24 (0.08) ^e^	3.49 (0.09) ^f^
SWE (mL_swollen_/g_dry solid_)	2.347 (0.003) ^e^	4.2 (0.3) ^c^	3.97 (0.08) ^cd^	3.72 (0.06) ^d^	5.12 (0.09) ^b^	5.58 (0.12) ^a^	5.21 (0.09) ^b^
Hy (g_w_/100 g_dry solid_)	19.7 (0.2) ^d^	21.5 (0.2) ^c^	22.4 (0.3) ^b^	22.42 (0.09) ^a^	22.73 (0.03) ^a^	22.7 (0.7) ^a^	22.6 (0.2) ^a^
SEI	14.6 (0.3) ^a^	10.7 (0.4) ^b^	9.8 (0.3) ^c^	8.6 (0.3) ^d^	8.3 (0.3) ^e^	7.3 (0.4) ^f^	6.9 (0.2) ^g^
ρ_b_ (g/cm^3^)	0.092 (0.008) ^d^	0.089 (0.002) ^d^	0.1137 (0.0004) ^bc^	0.1095 (0.0012) ^c^	0.118 (0.005) ^bc^	0.123 (0.009) ^b^	0.154 (0.006) ^a^
ε (%)	93.0 (0.6) ^a^	88.9 (0.3) ^b^	86.92 (0.06) ^c^	86.35 (0.12) ^cd^	85.3 (0.9) ^d^	85.5(0.9) ^d^	85.2 (0.6) ^d^
W_c_ (N*mm)	0.23 (0.03) ^c^	0.179 (0.009) ^c^	0.22 (0.04) ^c^	0.23 (0.05) ^c^	0.25 (0.04) ^c^	0.49 (0.09) ^b^	0.6 (0.2) ^a^
N_sr_ (mm^−1^)	9.69 (1.05) ^cd^	11.5 (0.8) ^a^	10.2 (0.9) ^bc^	11.9 (0.7) ^a^	11.1 (0.9) ^ab^	9.5 (0.6) ^cd^	9.0 (1.2) ^d^
F_s_ (N)	2.5 (0.3) ^c^	2.1 (0.2) ^c^	2.46 (0.3) ^c^	2.8 (0.5) ^c^	2.8 (0.4) ^c^	4.6 (0.9) ^b^	5.4 (0.9) ^a^
F_p_ (N)	2.0 (0.3) ^cd^	1.8 (0.2) ^d^	2.0 (0.2) ^cd^	2.4 (0.4) ^cd^	2.4 (0.4) ^c^	3.9 (0.8) ^b^	4.7 (0.8) ^a^
N_0_	108 (8) ^ab^	121 (5) ^a^	103 (12) ^b^	114 (9) ^ab^	111 (12) ^ab^	81 (7) ^c^	72 (13) ^c^

The same letter in superscript within a row indicates homogeneous groups established by ANOVA (*p <* 0.05). All data represent the mean of three determinations.

**Table 2:** Pearson correlation coefficients among studied parameters of extruded product.

	a_w_	WAI	WSI	SWE	Hy	SEI	ρ_b_	ε	W_c_	N_sr_	F_s_	F_p_	N_0_	MS Percentages
x_w_	0.7330 *	−0.8013 *	0.8127 *	−0.8488 *	−0.8208 *	0.9155 *	−0.7168 *	0.8948 *	−0.5529 *	0.0425	−0.6385 *	−0.6642 *	0.4909	−0.8202 *
a_w_		−0.8915 *	0.9394 *	−0.7945 *	−0.6591 *	0.8300 *	−0.8092 *	0.7713 *	−0.7389 *	0.2940	−0.8174 *	−0.8368 *	0.6841 *	−0.9522 *
WAI			−0.9803 *	0.7444 *	0.8272 *	−0.9212 *	0.8472 *	−0.8701 *	0.6758 *	−0.1314	0.7534 *	0.7908 *	−0.5958 *	0.9447 *
WSI				−0.7894 *	−0.8118 *	−0.9284 *	−0.8676 *	0.8793 *	−0.7075 *	0.2238	−0.7842 *	−0.8129 *	0.6669 *	−0.9784 *
SWE					0.8161 *	−0.8994 *	0.6702 *	−0.8658 *	0.5744 *	−0.1660	0.6427 *	0.6730 *	−0.5475 *	0.8597 *
Hy						−0.9314 *	0.6092 *	−0.9426 *	0.3322	0.0704	0.4100	0.4528	-0.3476	0.7855 *
SEI							−0.7667 *	0.9723 *	−0.5647 *	0.0588	−0.6519 *	−0.6898 *	0.5339 *	−0.9202 *
ρ_b_								−0.7355 *	0.8709 *	−0.5390 *	0.8865 *	0.8963 *	−0.8303 *	0.8995 *
ε									−0.4637	−0.0154	−0.5509 *	−0.5877 *	0.4272	−0.8599 *
W_c_										−0.7482 *	0.9854 *	0.9758 *	−0.9455 *	0.7869 *
N_sr_											−0.6552 *	−0.6084 *	0.8526 *	−0.3470
F_s_												0.9966 *	−0.9222 *	0.8494 *
F_p_													−0.9027 *	0.8731 *
N_0_														−0.7549 *

* Correlation is significant at 0.05. All data represent the mean of three determinations.

**Table 3:** Mean values (and standard deviations) of color coordinates (L*, a*, b*, C*, and h*) and total color differences (ΔE) of corn mixtures and extrudates with 0–15% MS.

Parameter	0	2.5	5	7.5	10	12.5	15
	**Mixtures**						
L*	80.5 (0.5) ^aA^	71.4 (0.4) ^bA^	68.4 (0.7) ^cA^	66.14 (0.02) ^dA^	63.0 (0.3) ^eA^	59.1 (0.6) ^eA^	60.5 (1.3) ^gA^
a*	6.3 (0.7) ^aA^	0.0 (0.2) ^bB^	−1.7 (0.3) ^cB^	−1.68 (0.15) ^cB^	−2.4 (0.2) ^dB^	−3.03 (0.07) ^eB^	−3.1 (0.3) ^eB^
b*	41.5 (1.7) ^aA^	33.0 (1.5) ^bA^	29.9 (0.4) ^cdA^	31.0 (0.6) ^cA^	31.0 (0.8) ^cA^	29.2 (0.5) ^dA^	29.6 (0.5) ^cdA^
C	41.9 (1.8) ^aA^	33.0 (1.5) ^bA^	30.0 (0.4) ^cdA^	31.0 (0.6) ^cA^	31.1 (0.8) ^cA^	29.4 (0.5) ^dA^	29.8 (0.5) ^cdA^
h	81.4 (0.6) ^eB^	90.0 (0.4) ^dA^	93.3 (0.6) ^cA^	93.1 (0.3) ^cA^	94.4 (0.4) ^bA^	95.9 (0.2) ^aA^	96.0 (0.6) ^aA^
ΔE_1_	−	18 (3) ^aA^	15.3 (0.4) ^bA^	15.2 (0.5) ^bA^	14.4 (0.7) ^bcA^	12.6 (0.5) ^cA^	13 (0.4) ^cA^
	**Extrudates**						
L*	60.3 (0.9) ^aB^	59.51 (1.12) ^aB^	59 (3) ^abB^	56 (3) ^bcB^	53.8 (1.2) ^dB^	54.9 (1.4) ^cdB^	54.2 (0.9) ^cdB^
a*	0.3 (0.4) ^bcB^	0.7 (0.2) ^aA^	0.72 (0.12) ^aA^	0.4 (0.2) ^bA^	0.28 (0.09) ^bcA^	−0.5 (0.2) ^dA^	0.07 (0.12) ^cA^
b*	17.1 (0.7) ^dB^	21.9 (0.7) ^cB^	23.6 (0.9) ^bB^	24.8 (1.3) ^aB^	22.9 (0.4) ^bB^	25.3 (0.6) ^aB^	25.4 (0.7) ^aB^
C	17.1 (0.7) ^dB^	21.9 (0.7) ^cB^	23.6 (0.9) ^bB^	24.8 (1.3) ^aB^	22.9 (0.4) ^bB^	25.3 (0.6) ^aB^	25.4 (0.7) ^aB^
h	89.0 (1.2) ^cA^	88.2 (0.4) ^eB^	88.3 (0.3)^dB^	89.0 (0.5) ^cB^	89.3 (0.2) ^bcB^	91.3 (0.5) ^aB^	89.8 (0.3) ^bB^
ΔE_1_	−	4.9 (0.6) ^eB^	7.2 (0.9) ^dB^	9.2 (0.8) ^bcB^	8.7 (0.8) ^cB^	9.8 (0.9) ^abB^	10.3 (0.9) ^aB^
ΔE_2_	32.21 (0.12) ^a^	16.3 (1.3) ^b^	12 (3) ^c^	12 (4) ^c^	12.63 (1.12) ^c^	6.3 (0.9) ^d^	8.3 (0.9) ^d^

For each parameter, the same small letter in superscript within a row indicates homogeneous groups established by ANOVA (*p <* 0.05) comparing MS percentages (0, 2.5, 5, 7.5, 10, 12.5, and 15) in mixtures or extrudates. For each MS percentages (0, 2.5, 5, 7.5, 10, 12.5, and 15) and parameter, the same capital letter in superscript within a column indicates homogeneous groups established by ANOVA (*p <* 0.05) comparing mixtures and extrudates. L*(lightness), a* (red/green coordinate), b* (yellow/blue coordinate), C* (chroma), and h* (tone). All data represent the mean of three determinations.

**Table 4:** Mean values (and standard deviations) of phenolic acids (caff (caffeic acid), Sir (Siringic acid), p-Coum (p-Coumaric acid), Fer (Ferulic acid), Di-caff (Di-caffeoylquinic acid)), folates, and antioxidant capacity (AC) content (µg/g_dry weight_) of corn mixtures and extrudates with 0–15% MS.

Compounds	0	2.5	5	7.5	10	12.5	15
	**Mixtures**						
Caff	18.53 (0.08) ^aB^	17.83 (0.04) ^bB^	17.44 (0.17) ^cA^	16.82 (0.05) ^dB^	16.58 (0.08) ^dB^	16.10 (0.05) ^eB^	15.79 (0.16) ^fB^
Sir	13.74 (0.07) ^aA^	13.45 (0.03) ^bA^	13.19 (0.02) ^bA^	12.84 (0.02) ^cA^	12.75 (0.15) ^cA^	12.07 (0.02) ^dA^	11.5 (0.22) ^eA^
p-coum	63.52 (0.28) ^aA^	60.83 (0.02) ^bA^	60.06 (0.09) ^cA^	58.55 (0.09) ^dA^	57.8 (0.21) ^eA^	55.1 (0.2) ^fA^	52.98 (0.14) ^gA^
Fer	31.37 (0.23) ^aB^	29.84 (0.12) ^bB^	29.09 (0.19) ^cB^	28.56 (0.02) ^dB^	28.32 (0.12) ^dB^	26.99 (0.16) ^eB^	26.22 (0.09) ^fB^
Di-caff	47.06 (0.12) ^aB^	45.36 (0.07) ^bB^	44.22 (0.18) ^cB^	42.93 (0.12) ^dB^	42.19 (0.02) ^eB^	41.08 (0.14) ^fB^	39.6 (0.21) ^gB^
Folates	0.83 (0.04) ^gA^	1.35 (0.04) ^fA^	2.05 (0.05) ^eA^	2.66 (0.03) ^dA^	3.39 (0.02) ^cA^	4.28 (0.07) ^bA^	4.86 (0.02) ^aA^
AC (TEq)	124 (18) ^gA^	169 (5) ^fA^	196 (5) ^eA^	228 (5) ^dA^	262 (5) ^cA^	320 (5) ^bA^	358 (9) ^aA^
	**Extrudates**						
Caff	20.05 (0.02) ^aA^	18.26 (0.05) ^bA^	17.82 (0.05) ^cA^	17.69 (0.02) ^cA^	17.50 (0.06) ^dA^	16.96 (0.12) ^eA^	16.63 (0.12) ^fA^
Sir	12.26 (0.03) ^aB^	11.85 (0.15) ^aB^	11.69 (0.12) ^aB^	12.0 (0.7) ^abA^	11.0 (0.2) ^bcB^	10.66 (0.09) ^cB^	10.42 (0.09) ^cB^
p-coum	60.02 (0.08) ^aB^	57.55 (0.06) ^bB^	56.88 (0.14) ^cB^	55.54 (0.04) ^dB^	54.13 (0.02) ^eB^	51.80 (0.17) ^fB^	49.67 (0.15) ^gA^
Fer	40.44 (0.15) ^aA^	38.74 (0.07) ^bA^	37.83 (0.12) ^cA^	36.5 (0.5) ^dA^	35.46 (0.07) ^eA^	34.24 (0.14) ^fA^	33.55 (0.12) ^gA^
Di-caff	57.22 (0.07) ^aA^	54.57 (0.14) ^bA^	53.61 (0.08) ^cA^	51.87 (0.14) ^dA^	50.23 (0.05) ^eA^	48.77 (0.12) ^fA^	47.65 (0.08) ^gA^
Folates	0.64 (0.02) ^gB^	1.00 (0.03) ^fB^	1.27 (0.08) ^eB^	1.72 (0.20) ^dB^	1.96 (0.04) ^cB^	2.80 (0.05) ^bB^	4.18 (0.18) ^aB^
AC (TEq)	^−gB^	161 (4) ^fA^	180 (4) ^eA^	198 (4) ^dB^	216 (4) ^cB^	241 (4) ^bB^	257 (4) ^aB^

For each compound, the same small letter in superscript within a row indicates homogeneous groups established by ANOVA (*p <* 0.05) comparing MS % (0, 2.5, 5, 7.5, 10, 12.5, and 15) in mixtures or extrudates. For each MS % (0, 2.5, 5, 7.5, 10, 12.5, or 15) and compound, the same capital letter in superscript within a column indicates homogeneous groups established by ANOVA (*p <* 0.05) comparing mixtures and extrudates. All data represent the mean of three determinations.

**Table 5:** Mean values (and standard deviations) of flavonoids A-gluc (Apigenin-glucoside), A-glucur (Apigenin-glucuronide), A-diglucur (Apigenin-diglucuronide), L-fer-ggg (Luteolin-(feruloyl-glucuronyl-glucuronyl)-glucuronide), A-fer-gg (Apigenin-(feruloyl-glucuronyl)-glucuronide), A-fer-ggg (Apigenin-(feruloyl-glucuronyl-glucuronyl)-glucuronide), L-diglucur (Luteolin-diglucuronide), Liq-gen (Liquirtigenin), D-gluc (Daidzein-glucoside), G-gluc (Genistein-glucoside), D (Daidzein), and G (Genistein) content (µg/g_dry weight_) of *Medicago sativa,* corn mixtures, and extrudates with 0–15% MS.

Flavonoids		MS	0	2.5	5	7.5	10	12.5	15
			**Mixtures**						
*Flavones*	A-gluc	219.15 (0.05)	^−^^d^	^−^^d^	^−^^d^	^−^^dA^	5.01 (0.02) ^cA^	14.15 (0.06) ^bA^	28.79 (0.04) ^aA^
	A-glucur	669.39 (0.02)	^−^^f^	^−^^f^	5.432 (0.013) ^eA^	10.85(0.04) ^dA^	23.22 (0.03) ^cA^	25.12 (0.03) ^bA^	46.14 (0.08) ^aA^
	A-diglucur	999.907 (0.015)	^−^^f^	^−^^f^	7.225 (0.015) ^eA^	15.79 (0.05) ^dA^	36.61 (0.06) ^cA^	46.21 (0.08) ^bA^	62.65 (0.04) ^aA^
	L-fer-ggg	1,543.88 (0.08)	^−^^g^	8.50 (0.08) ^fA^	24.97 (0.13) ^eA^	38.32 (0.04) ^dA^	51.27 (0.04) ^cA^	58.93 (0.06) ^bA^	72.73 (0.08) ^aA^
	A-fer-gg	317.00 (0.03)	^−^^g^	31.073 (0.014) ^fA^	68.243 (0.015) ^eA^	125.25(0.03) ^dA^	257.56 (0.03) ^cA^	386.85 (0.08) ^bA^	552.06 (0.27) ^aA^
	A-fer-ggg	1,075.27 (0.17)	^−^^g^	36.22 (0.08) ^fA^	38.10 (0.17) ^eA^	51.453 (0.104) ^dA^	64.20 (0.08) ^cA^	68.52 (0.11) ^bA^	77.90 (0.04) ^aA^
	L-diglucur	840.53 (0.04)	^−^^g^	37.54 (0.04) ^fA^	48.25 (0.09) ^eA^	55.26 (0.08) ^dA^	62.23 (0.04) ^cA^	62.78 (0.10) ^bA^	74.77 (0.15) ^aA^
	Liq-gen	534.5 (0.3)	^−^^g^	29.15 (0.13) ^fA^	35.57 (0.13) ^eA^	43.55 (0.39) ^dA^	57.56 (0.18) ^cA^	59.84 (0.45) ^bA^	62.17 (0.27) ^aA^
*Isoflavones*	D-gluc	618.84 (0.05)	^−^^g^	2.57 (0.06) ^fA^	4.915 (0.016) ^eA^	15.17 (0.07) ^dA^	18.61 (0.08) ^cA^	31.48 (0.03) ^bA^	53.67 (0.06) ^aA^
	G-gluc	1,000.634 (0.013)	^−^^f^	^−^^f^	22.46 (0.09) ^eA^	33.34 (0.21) ^dA^	55.182 (0.105) ^cA^	56.99 (0.02) ^bA^	69.45 (0.17) ^aA^
	D	607.42 (0.03)	^−^^g^	22.69 (0.13) ^fA^	31.67 (0.17) ^eA^	40.16 (0.38) ^dA^	48.71 (0.06) ^cA^	55.75 (0.06) ^bA^	68.41 (0.13) ^aA^
	G	186.51 (0.07)	^−^^c^	^−^^c^	^−^^c^	^−^^c^	^−^^c^	4.32 (0.08) ^bA^	9.86 (0.03) ^aA^
			**Extrudates**						
*Flavones*	A-gluc		−	−	−	^−^	^−^^B^	^−^^B^	^−^^B^
	A-glucur		−	−	^−^^B^	^−^^B^	^−^^B^	^−^^B^	^−^^B^
	A-diglucur		−	−	^−^^B^	^−^^B^	^−^^B^	−	^−^^B^
	L-fer-ggg		^−^^g^	2.16 (0.05) ^fB^	4.20 (0.05) ^eB^	5.01 (0.06) ^dB^	12.89 (0.02) ^cB^	15.59 (0.05) ^bB^	17.18 (0.08) ^aB^
	A-fer-gg		^−^^g^	3.65 (0.02) ^fB^	6.35 (0.08) ^eB^	12.87 (0.07) ^dB^	22.05 (0.05) ^cB^	29.72 (0.08) ^bB^	32.00 (0.13) ^aB^
	A-fer-ggg		^−^^g^	16.73 (0.05) ^fB^	21.69 (0.28) ^eB^	23.40 (0.15) ^dB^	25.75 (0.07) ^cB^	29.22 (0.13) ^bB^	32.79 (0.06) ^aB^
	L-diglucur		^−^^f^	0.65 (0.07) ^eB^	3.66 (0.04) ^dB^	3.91 (0.07) ^dB^	11.85 (0.26) ^cB^	20.16 (0.14) ^bB^	23.77 (0.05) ^aB^
	Liq-gen		^−^^g^	5.82 (0.09) ^fB^	11.32 (0.17) ^eB^	18.59 (0.20) ^dB^	21.66 (0.27) ^cB^	22.63 (0.16) ^bB^	23.93 (0.28) ^aB^
Flavonoids		**MS**	**0**	**2.5**	**5**	**7.5**	**10**	**12.5**	**15**
*Isoflavones*	D-gluc		^−^^f^	^−^^fB^	0.54 (0.07) ^eB^	1.07 (0.09) ^dB^	1.36 (0.02) ^cB^	2.04 (0.07) ^bB^	2.26 (0.06) ^aB^
	G-gluc		^−^^d^	^−^^d^	^−^^dB^	^−^^dB^	3.99 (0.05) ^cB^	4.571 (0.117) ^bB^	5.75 (0.06) ^aB^
	D		^−^^g^	11.313 (0.117) ^fB^	11.99 (0.09) ^e^	13.62 (0.03) ^dB^	17.36 (0.18) ^cB^	21.45 (0.19) ^bB^	24.46 (0.17) ^aB^
	G		−	−	−	−	−	^−^^B^	^−^^B^

For each compound, the same small letter in superscript within a row indicates homogeneous groups established by ANOVA (*p <* 0.05) comparing MS % (0, 2.5, 5, 7.5, 10, 12.5, and 15) in mixtures or extrudates. For each MS % (0, 2.5, 5, 7.5, 10, 12.5, or 15) and compound, the same capital letter in superscript within a column indicates homogeneous groups established by ANOVA (*p <* 0.05) comparing mixtures and extrudates. All data represent the mean of three determinations.

**Table 6:** Mean values (and standard deviation) of total peak area percentage of MS aroma compounds. All data represent the mean of three determinations.

Volatile Compounds		Odor Perception	MS
*Alcohols*	1-Pentanol	fruity	0.28 (0.02)
	3-methyl-1-butanol	whiskey, malt, burnt	0.38 (0.05)
	2-methyl-1 butanol	malt	0.45 (0.02)
	1-Hexanol	ethereal, oil, alcohol, green, fruity, sweet, woody, floral	0.12 (0.03)
*Aldehydes*	Benzaldehyde	almond, burnt sugar	0.17 (0.03)
	Hexanal	fresh, green, fatty, aldehydic, grass, leafy	0.65 (0.21)
	heptanal	fat, citrus	
	2-Octenal, (E)-	green, nut	
	2-Hexenal, (E)-	green, leaf	0.12 (0.02)
	Nonanal	fat, citrus, green	
*Terpenes*	β -Pinene	pine, resin, turpentine	1.95 (0.07)
*and*	α-Pinene	pine, turpentine	5.47 (0.12)
*terpenoids*	β-Myrcene	balsamic, must, spice	21.78 (0.15)
	3-Carene	lemon, resin	1.56 (0.07)
	*p*-Cymene	citrus, sweet, herbal	8.57 (0.03)
	Limonene	citrus, mint	45.34 (0.04)
	Eucalyptol	mint, sweet	3.28 (0.08)
	Camphene	camphor	0.65 (0.04)
	γ-Terpinene	lemon	2.05 (0.09)
	Terpinolene	fresh, woody, sweet, pine, citrus	0.47 (0.03)
	Sabinene	pepper, turpentine, wood	1.66 (0.08)
	Caryophyllene	wood, spice	0.06 (0.02)
	Cyclohexanone, 5-methyl-2-(1-methylethyl)-,(2R-cis)-	minty	0.24 (0.03)
	α-Thujene	wood, green, herb	3.03 (0.03)
	α-Terpinene	lemon	0.61 (0.07)
	β-Linalool	flower, lavender	0.04 (0.08)
	γ-Terpinene	lemon	
	beta cis Ocimene	citrus, herb, flower	0.1 (0.02)
	beta trans Ocimene	sweet, herb	0.14 (0.03)
*Acids*	Benzoic acid	balsamic	0.19 (0.05)
*Ketones*	3-Pentanone, 2,4-dimethyl-	-	0.09 (0.07)
	3-Octanone	herbal, sweet, mushroom	0.49 (0.03)
	2,2,6-trimethyl-Cyclohexanone	thujonic	0.06 (0.02)
*Hydrocarbons*	Toluene	paint	0.13 (0.02)
*Esters*	Propanoic acid, 2-methyl-, 2-methylbutyl ester -	fruity ethereal tropical banana	0.17 (0.05)
	Octanoic acid, ethyl ester	fruit, fat	0.86 (0.02)
	Butanoic acid, methyl ester	ether, fruit, sweet	0.12 (0.01)
	Propanoic acid, 2-methyl-, 2-methylpropyl ester	fruity	0.08 (0.02)

**Table 7:** Mean values (and standard deviation) of total peak area percentage of aroma compounds from mixtures and extrudates with 0–15% MS.

Volatile Compounds		0	2.5	5	7.5	10	12.5	15
	**Mixtures**							
*Alcohols*	1-Pentanol	0.35 (0.03) ^eA^	0.55 (0.03) ^bA^	0.42 (0.02) ^cdA^	0.38 (0.04) ^deA^	0.27 (0.02) ^fA^	0.47 (0.05) ^cB^	0.8 (0.03) ^aB^
	3-methyl-1-butanol	^−e^	^−e^	0.44 (0.04) ^d^	0.72 (0.01) ^b^	0.44 (0.01) ^d^	0.66 (0.01) ^c^	0.77 (0.02) ^a^
	2-methyl-1 butanol	^−f^	^−f^	0.28 (0.03) ^e^	0.54 (0.02) ^c^	0.41 (0.02) ^d^	0.71 (0.02) ^b^	0.83 (0.05) ^a^
	1-Hexanol	0.18 (0.02) ^f^	0.28 (0.03) ^d^	0.14 (0.02) ^g^	0.24 (0.02) ^e^	0.55 (0.02) ^a^	0.3 (0.05) ^c^	0.5 (0.03) ^b^
*Aldehydes*	Benzaldehyde	^−e^	0.3 (0.02) ^b^	^−e^	^−e^	0.24 (0.02) ^d^	0.48 (0.12) ^a^	0.26 (0.03) ^c^
	Hexanal	4.23 (0.16) ^cB^	5.27 (0.04) ^aB^	4.92 (0.09) ^abB^	4.83 (0.08) ^bB^	3.65 (0.23) ^dB^	2.95 (0.12) ^eB^	2.00 (0.45) ^fB^
	β-Pinene	3.98 (0.04) ^aA^	3.4 (0.03) ^bA^	2.83 (0.33) ^cA^	2.31 (0.05) ^dA^	1.89 (0.03) ^eA^	1.50 (0.22) ^fA^	0.89 (0.17) ^gB^
	α-Pinene	9.29 (0.03) ^aA^	6.77 (0.05) ^bA^	6.14 (0.18) ^cA^	5.84 (0.22) ^cA^	4.45 (0.15) ^dA^	3.71 (0.28) ^eA^	2.44 (0.28) ^fA^
	β-Myrcene	12.42(0.16) ^gA^	14.79 (0.32) ^fA^	16.70 (0.56) ^eA^	18.94 (0.77) ^dA^	22.00 (0.93) ^cA^	23.30 (0.16) ^bA^	25.10 (0.05) ^aA^
	3-Carene	1.95 (0.03) ^a^	1.77 (0.09) ^b^	1.93 (0.08) ^ab^	1.87 (0.08) ^ab^	1.89 (0.03) ^ab^	1.8 (0.07) ^ab^	1.83 (0.08) ^ab^
	*p*-Cymene	7.45 (0.16) ^eA^	7.91 (0.34) ^dA^	8.21 (0.44) ^dA^	8.71 (0.44) ^cA^	9.26 (0.76) ^bA^	9.89 (0.05) ^aA^	10.21 (0.09) ^a^
	Limonene	23.66 (0.51) ^eA^	25.15 (0.34) ^deA^	28.04 (0.55) ^cdA^	30.49 (0.56) ^bcA^	33.02 (0.56) ^bA^	36.49 (0.39) ^aA^	38.85 (0.79) ^aA^
	Eucalyptol	14.7 (0.13) ^aA^	13.73 (0.2) ^bA^	12.30 (0.45) ^cA^	11.63 (0.77) ^dA^	10.86 (0.37) ^eA^	9.43 (0.79) ^fA^	8.42 (0.88) ^gA^
	Camphene	1.81 (0.03) ^a^	1.50 (0.03) ^b^	1.31 (0.03) ^c^	1.17 (0.08) ^d^	1.03 (0.02) ^d^	0.86(0.33) ^e^	0.75 (0.21) ^e^
	γ-Terpinene	1.17 (0.09) ^fA^	1.41 (0.07) ^cA^	1.47 (0.05) ^bA^	1.10 (0.07) ^gA^	1.30 (0.06) ^eA^	1.36 (0.09) ^dB^	1.68 (0.07) ^aA^
	Sabinene	1.47 (0.03) ^bc^	1.53 (0.04) ^bc^	1.62 (0.04) ^b^	1.5 (0.02) ^bc^	1.38 (0.04) ^cd^	1.88 (0.16) ^a^	1.26 (0.07) ^d^
	α-Thujene	1.96 (0.05) ^abB^	1.56 (0.05) ^cA^	1.87 (0.02) ^bA^	1.6 (0.05) ^cA^	1.69 (0.03) ^cA^	2.08 (0.02) ^aA^	1.63 (0.18) ^cA^
	α-Terpinene	0.45 (0.03) ^a^	^−^b	^−^b	^−^b	^−^b	^−^b	^−^b
	β-Linalool	1.23 (0.02) ^aA^	1.06 (0.33) ^bA^	0.86 (0.12) ^cA^	0.83 (0.2) ^cdA^	0.71 (0.12) ^dA^	0.47 (0.21) ^eA^	0.34 (0.02) ^fA^
*Ketones*	3-Octanone	0.84 (0.04) ^a^	0.7 (0.02) ^b^	^−c^	^−c^	^−c^	^−c^	^−c^
*Hydrocarbons*	Toluene	0.96 (0.05) ^aB^	0.4 (0.03) ^dB^	0.59 (0.03) ^bB^	0.5 (0.04) ^cB^	0.52 (0.04) ^cB^	0.34 (0.02) ^dB^	0.4 (0.03) ^dB^
*Esters*	Octanoic acid, ethyl ester	1.20 (0.07) ^cB^	1.01 (0.04) ^eB^	0.71 (0.02) ^fB^	1.88 (0.03) ^aB^	0.65 (0.02) ^gB^	1.09 (0.04) ^dA^	1.79 (0.03) ^bA^
	Butanoic acid, methyl ester	0.15 (0.04) ^a^	^−b^	^−b^	^−b^	^−b^	^−b^	^−b^
	**Extrudates**							
*Alcohols*	1-Pentanol	^−cB^	^−cB^	^−cB^	^−cB^	^−cB^	0.75 (0.05) ^bA^	0.99 (0.08) ^aA^
	Hexanal	23.86 (0.03) ^gA^	26.72 (0.07) ^fA^	29.73 (0.55) ^eA^	32.16 (0.37) ^dA^	34.43 (0.53) ^cA^	36.23 (0.06^)^^bA^	38.21 (0.59) ^aA^
	Heptanal	^−d^	^−d^	^−d^	^−d^	1.01 (0.02) ^b^	1.99 (0.02) ^a^	0.7 (0.07) ^c^
	2-Octenal, (E)-	^−c^	^−c^	^−c^	^−c^	1.83 (0.18) ^a^	1.1 (0.03) ^b^	0.44 (0.02) ^c^
	Nonanal	^−d^	^−d^	^−d^	^−d^	2.78 (0.05) ^b^	5.9 (0.07) ^a^	1.0 (0.06) ^c^
*Terpenes*	β -Pinene	^−dB^	^−dB^	2.07 (0.03) ^aB^	^−dB^	1.22 (0.04) ^bB^	1.00 (0.07) ^cB^	1.28 (0.03) ^bB^
	α-Pinene	5.03 (0.37)^a B^	4.04 (0.07) ^bB^	3.19 (0.33) ^cB^	2.89 (0.7) ^dB^	2.06 (0.88) ^eB^	1.10 (0.55) ^fB^	0.61 (0.08) ^gB^
	β-Myrcene	11.19 (0.22) ^gB^	12.73 (0.55) ^fB^	13.67 (0.08) ^eB^	16.09 (0.05) ^dB^	17.61 (0.88) ^cB^	18.99 (0.06) ^bB^	21.07 (0.65) ^aB^
	*p*-Cymene	2.83 (0.06) ^fB^	3.11 (0.43) ^eB^	3.55 (0.88) ^dB^	4.01 (0.55) ^cB^	4.42 (0.39) ^bB^	4.93 (0.56) ^aB^	5.02 (0.52) ^aB^
	Limonene	19.67 (0.86) ^gB^	20.90 (0.04) ^fB^	21.95 (0.55) ^eB^	23.48 (0.34) ^dB^	25.40 (0.39) ^cB^	27.21 (0.47) ^bB^	29.17 (0.76) ^aB^
	Eucalyptol	11.25 (0.07) ^a^	10.61 (0.23) ^aB^	9.13 (0.14) ^bB^	8.49(0.56) ^bB^	7.80 (0.08) ^cB^	6.75 (0.33) ^dB^	5.38 (0.64) ^eB^
	α-Thujene	4.16 (0.08) ^aA^	^−cB^	^−cB^	^−cB^	0.59 (0.03) ^bB^	^−cB^	^−cB^
	β-Linalool	1.14 (0.03) ^aA^	0.98 (0.21) ^abA^	0.88 (0.09) ^bcA^	0.81 (0.07) ^cA^	0.59 (0.04) ^dB^	0.39 (0.03) ^eA^	0.17 (0.07) ^fB^
	γ-Terpinene	^−dB^	^−dB^	^−dB^	^−dB^	0.42 (0.02) ^cB^	1.61 (0.03) ^aA^	1.54 (0.03) ^bB^
*Hydrocarbons*	Toluene	3.83 (0.08) ^aA^	2.19 (0.04) ^cA^	2.04 (0.04) ^dA^	2.63 (0.03) ^bA^	1.19 (0.03) ^fA^	1.61 (0.07) ^eA^	2.17 (0.06) ^cA^
*Esters*	Octanoic acid, ethyl ester	3.15 (0.05) ^cA^	4.33 (0.03) ^aA^	3.44 (0.16) ^bA^	2.03 (0.02) ^dA^	1.01 (0.03) ^eA^	0.39 (0.05) ^fB^	0.5 (0.02) ^fB^

For each compound, the same small letter in superscript within a row indicates homogeneous groups established by ANOVA (*p <* 0.05) comparing MS % (0, 2.5, 5, 7.5, 10, 12.5, and 15) in mixtures or extrudates. For each MS % (0, 2.5, 5, 7.5, 10, 12.5, or 15) and compound, the same capital letter in superscript within a column indicates homogeneous groups established by ANOVA (*p <* 0.05) comparing mixtures and extrudates. All data represent the mean of three determinations.
